# Study of Dependence of Kinetic Freezeout Temperature on the Production Cross-Section of Particles in Various Centrality Intervals in Au–Au and Pb–Pb Collisions at High Energies

**DOI:** 10.3390/e23040488

**Published:** 2021-04-20

**Authors:** Muhammad Waqas, Guang-Xiong Peng

**Affiliations:** 1 School of Nuclear Science and Technology, University of Chinese Academy of Sciences, Beijing 100049, China; waqas_phy313@yahoo.com or; 2 Theoretical Physics Center for Science Facilities, Institute of High Energy Physics, Beijing 100049, China

**Keywords:** non-strange, strange, multi-strange, kinetic freeze-out temperature, transverse flow velocity, freezeout volume, cross-section, centrality bins, transverse momentum spectra, 12.40.Ee, 13.85.Hd, 25.75.Ag, 25.75.Dw, 24.10.Pa

## Abstract

Transverse momentum spectra of $\pi^{+}$, *p*, Λ, Ξ or ${\bar{\Xi }}^{+}$, Ω or ${\bar{\Omega }}^{+}$ and deuteron (*d*) in different centrality intervals in nucleus–nucleus collisions at the center of mass energy are analyzed by the blast wave model with Boltzmann Gibbs statistics. We extracted the kinetic freezeout temperature, transverse flow velocity and kinetic freezeout volume from the transverse momentum spectra of the particles. It is observed that the non-strange and strange (multi-strange) particles freezeout separately due to different reaction cross-sections. While the freezeout volume and transverse flow velocity are mass dependent, they decrease with the resting mass of the particles. The present work reveals the scenario of a double kinetic freezeout in nucleus–nucleus collisions. Furthermore, the kinetic freezeout temperature and freezeout volume are larger in central collisions than peripheral collisions. However, the transverse flow velocity remains almost unchanged from central to peripheral collisions.

## 1. Introduction

Freezeout stages are very important because they provide essential information about the emissions of the particles at those stages. Generally, there are two freezeout stages found in the literature—namely, the chemical freezeout and kinetic freezeout stage—and both of these correspond to their respective temperatures. The chemical freezeout is the intermediate stage in high-energy collisions where the intra-nuclear collisions between the particles are inelastic and the ratio of various types of particles remain unchanged; the temperature of the particles at this stage is the chemical freezeout temperature, which describes the excitation degree of the system at the chemical freezeout stage. Correspondingly, the thermal/kinetic freezeout is the last stage in high-energy collisions. At this stage, the intra-nuclear collisions between the particles are elastic. The transverse momentum distributions of various kinds of particles are no longer changed at the thermal freezeout stage, and the temperature at this stage is called the kinetic freezeout temperature.

According to the thermal and statistical model [1,2,3,4], the chemical freezeout temperature ($T_{ch}$) in central nucleus–nucleus collisions increases with the increase of the collision energy from a few GeV to above 10 GeV and then saturates in an energy range of more than 12 GeV. At the Relativistic Heavy Ion Collider (RHIC) and Large Hadron Collider (LHC), the maximum $T_{ch}$ is 160 MeV, although there is a slight decrease from the energy of RHIC to LHC, but the situation of the kinetic freezeout temperature ($T_{0}$) is complex. At first, $T_{0}$ in central collisions increases with the collision energy increasing from a few GeV to above 10 GeV, but this tendency can either be saturated, decreasing or increasing. On the other hand, $T_{ch}$ in central nucleus–nucleus collisions is a little larger than in peripheral nucleus–nucleus collisions; however, there are three possible trends of $T_{0}$ from central to peripheral collisions, which are (1) $T_{0}$ increases from central to peripheral collisions, (2) $T_{0}$ decreases from central to peripheral collisions, and (3) $T_{0}$ remains constant from central to peripheral collisions. It is very important to search for the correct trend of $T_{0}$ with energy and centrality. Furthermore, there are different kinetic freezeout scenarios found in the literature, which include single, double, triple and multiple kinetic freezeout scenarios [5,6,7,8,9,10]. In the single kinetic freezeout scenario, one set of parameters is used for the strange, multi-strange and non-strange particles. In the double kinetic freezeout scenario, one set of parameters is used for strange (multi-strange) and another for non-strange particles; separate sets of parameters are used for strange, multi-strange and non-strange particles in the triple kinetic freezeout scenario. In contrast, in the multiple kinetic freezeout scenario, separate sets of parameters are used for each particle. The trend of transverse flow velocity ($\beta_{T}$) and freezeout volume (*V*) with energy is an increasing trend in most of the literature [6,11,12,13,14,15,16]. Most of the literature claims to show a decreasing (or invariant) trend of $\beta_{T}$ and *V* from central to peripheral collisions [10,15,16,17,18].

The transverse momentum spectra ($p_{T}$) of the particles are very important observable variables due to the fact that they provide essential information about the equilibrium dynamics and isotropy of the system in high-energy collisions [9]. In the present work, we analyze the $p_{T}$ spectra of $\pi^{+}$, *p*, Λ, Ξ (${\bar{\Xi }}^{+}$), Ω (${\bar{\Omega }}^{+}$) and deuteron (*d*) in nucleus–nucleus collisions at the center of mass energy.

The remainder of the paper consists of the method and formalism in Section 2 and results and discussion in Section 3, and the summary of our main observations and conclusions are presented in Section 4.

## 2. Method and Formalism

There are various models suggested for the extraction of $T_{0}$, *V* and $\beta_{T}$; e.g., the blast wave model with Boltzmann Gibbs statistics (BGBW) [19,20,21], the blast wave model with Tsallis statistics (TBW) [22,23,24], an alternative method by using Tsallis statistics [25,26,27,28,29,30,31] and an alternative method by using the blast wave model with Boltzmann Gibbs statistics [32,33,34,35,36,37]. In this work, we choose the blast wave model with Boltzmann Gibbs statistics, which is a phenomenological model and is used for the spectra of hadrons based on the flow of local thermal sources with global variables of temperature, volume and transverse flow velocity.

According to [38,39,40], the $p_{T}$ distribution of the BGBW can be written as

(1) $$\begin{array}{cc} f(p_{T})= & \frac{1}{N}\frac{dN}{dp_{T}}=C\frac{gV}{{\left(2\pi \right)}^{2}}p_{T}m_{T}\int_{0}^{R}rdr\\ \; & \times I_{0}\left[\frac{p_{T}\sinh\left(\rho_{1}\right)}{T_{0}}\right]K_{1}\left[\frac{m_{T}\cosh\left(\rho_{1}\right)}{T_{0}}\right], \end{array}$$

where *C* stands for the normalization constant, *g* represents the degeneracy factor of the particles, *V* is the freezeout volume, $m_{T}=\sqrt{p_{T}^{2}+m_{0}^{2}}$ is the transverse mass ($m_{0}$ is the resting mass of the particle), *r* is the radial coordinate, *R* is the maximum *r*, $\rho =\tanh^{-1}\left[\beta \left(r\right)\right]$ is the boost angle, $\beta \left(r\right)=\beta_{S}{\left(r/R\right)}^{n_{0}}$ is a self-similar flow profile, and $\beta_{S}$ is the flow velocity on the surface, as a mean of β(r), $\beta_{T}=\left(2/R^{2}\right)\int_{0}^{R}r\beta \left(r\right)dr=2\;\beta_{S}/\left(n_{0}+2\right)$ if $n_{0}$ = 2, $\beta_{T}$ = 0.5 $\beta_{S}$, because the maximum $\beta_{S}$ is 1c and the maximum value of $\beta_{T}$ is 0.5; however, if $n_{0}$ = 1, this will result in $\beta_{T}=\left(2/3\right)\;\beta_{S}$, and thus the maximum $\beta_{T}$ is (2/3)c. However, if $n_{0}$ is used as a free parameter [41], it increases the value of 854 by several times in terms of the number of free parameters. $I_{0}$ and $K_{1}$ are the Bessel-modified functions of the first and second kind, respectively.

Equation (1) is not sufficient for the description of all $p_{T}$ spectra, particularly when the maximum $p_{T}$ reaches 100 GeV/c for collisions at the LHC [42], where several $p_{T}$ regions [43] have been observed by the model analysis. These regions include the first $p_{T}$ region with $p_{T}$ < 4.5 GeV/c, the second and third region with 4–6 GeV/c <$p_{T}$ < 17–20 GeV/c and $p_{T}$ > 17–20 GeV/c, respectively. It is expected that different $p_{T}$ regions correspond to different interaction mechanisms, such as the effects and changes according to the medium, nuclear transparency and the effect of the number of strings etc., which are discussed in detail in [17]. Therefore, for the complete description of the entire $p_{T}$, we can use functions such as Tsallis Levy [44,45] and the Hagedorn function [42,46,47] for the spectra in high and very high $p_{T}$ regions, and this corresponds to the inverse power law. In this work, we used the inverse power law to describe the $p_{T}$ spectra in high $p_{T}$ regions; that is,

(2) $$\begin{array}{cc} f_{H}\left(p_{T}\right)= & \frac{1}{N}\frac{dN}{dp_{T}}=Ap_{T}{\left(1+\frac{p_{T}}{p_{0}}\right)}^{-n}, \end{array}$$

where *N* and *A* represents the number of particles and normalization constant, respectively, and $p_{0}$ and *n* are the free parameters. There are several modified versions of the Hagedorn function found in the literature [48,49,50,51,52,53,54].

Generally, the two main processes responsible for the contribution of $p_{T}$ spectra are soft excitation (which contributes the soft component in the low $p_{T}$ region) and the hard scattering process (which contributes over the whole $p_{T}$ region). Equation (1) is taken into account for the soft excitation process and Equation (2) for the hard scattering process. Equations (1) and (2) can be superposed by two methods; i.e., (1) the super position principle, where the contribution regions of components overlap each other, and (2) the Hagedorn model (usual step function), when there is no overlapping of different regions of different components. According to the first method,

(3) $$\begin{array}{c} f_{0}\left(p_{T}\right)=kf_{S}\left(p_{T}\right)+\left(1-k\right)f_{H}\left(p_{T}\right), \end{array}$$

where *k* represents the contribution fraction of the first component and (1−k) represents the contribution function of the second component.

The usual step function can be used to structure the superposition of Equations (1) and (2). According to Hagedorn model [42,46,47], the usual step function can also be used for the superposition of the two functions, as

(4) $$\begin{array}{c} f_{0}\left(p_{T}\right)=A_{1}\theta \left(p_{1}-p_{T}\right)f_{1}\left(p_{T}\right)+A_{2}\theta \left(p_{T}-p_{1}\right)f_{2}\left(p_{T}\right), \end{array}$$

where $A_{1}$ and $A_{2}$ are the fraction constants which give the two components to be equal to each other at $p_{T}$ = $p_{1}$.

It should be noted that the soft and hard components in Equations (3) and (4) are treated in different ways over the whole $p_{T}$ region. Equation (3) is used for the contribution of the soft component in the range 0–2∼3 GeV/c or a little more. However, in the case of the contribution of the hard component, even though the main contribution in the low $p_{T}$ region is the soft excitation process, it covers the whole $p_{T}$ region. In Equation (4), in the range from 0 to $p_{1}$ and from $p_{1}$ up to the maximum, the contributions of the soft and hard components are present, respectively, and there is no mixed region for the two components. In addition, we would like to point out that, in the present work, we have used Equation (1) (which is a singl-component BGBW) only, but Equations (3) and (4) are stated in order to present the entire methodology and treatment (if Equation (2) is used). If we were to use a double-component BGBW, then we could use either Equations (3) or (4) to combine the two components.

## 3. Results and Discussion

**Figure 1:**
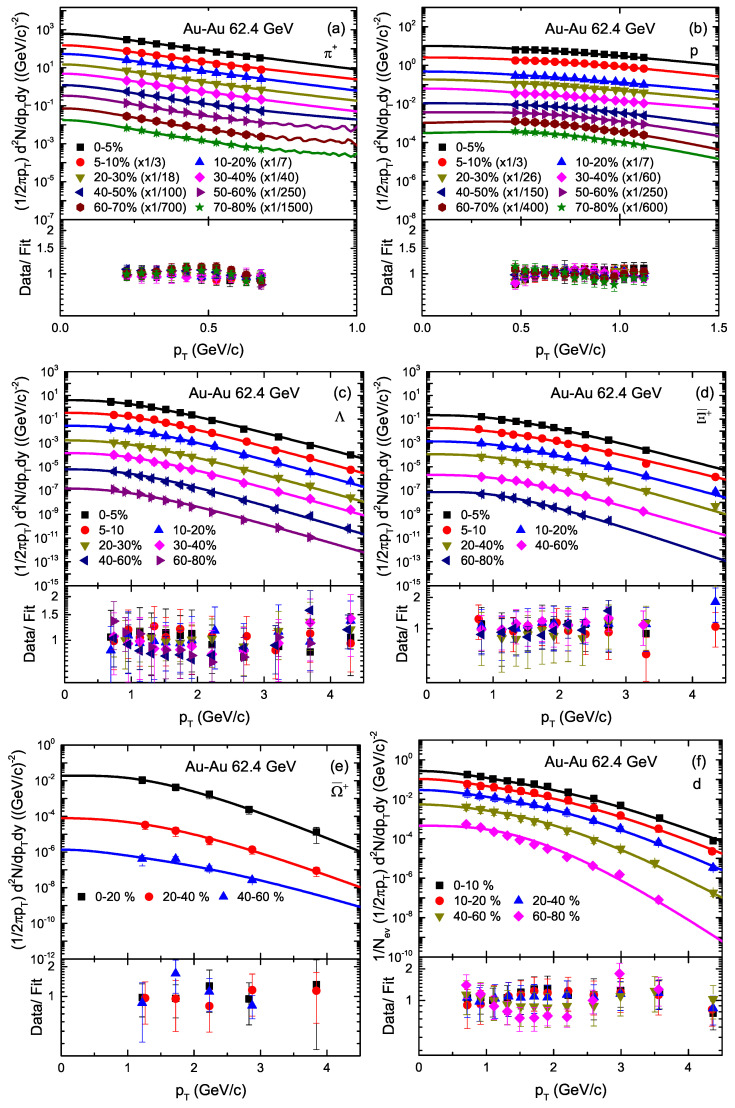
Transverse momentum spectra of $\pi^{+}$, *p*, Λ, ${\bar{\Xi }}^{+}$ and ${\bar{\Omega }}^{+}$ rapidity at |y|<0.1, and deuteron (d) at rapidity |y|<0.3, produced in different centrality intervals in Au–Au collisions at 62.4 GeV. Different symbols represent the $p_{T}$ spectra of different particles measured by the STAR collaboration [21,55,56] and the curves are our fitted results with the blast wave model with Boltzmann Gibbs statistics (BGBW). The corresponding results of the data/fit are presented in each panel.

Figure 1 demonstrates the transverse momentum ($p_{T}$) spectra, [(1/2π$p_{T}$) $d^{2}$*N*/dyd$p_{T}$] or [$1/N_{ev}$(1/2π$p_{T}$) $d^{2}$*N*/dyd$p_{T}$] of $\pi^{+}$, *p*, Λ, ${\bar{\Xi }}^{+}$, ${\bar{\Omega }}^{+}$ and deuteron (d) in various centrality classes in Au–Au collisions at 62.4 GeV. The spectra are distributed in different centrality classes; e.g., for $\pi^{+}$ and *p*, 0–5%, 5–10%, 10–20%, 20–30%, 30–40%, 40–50%, 50–60%, 60–70% and 70–80%, for Λ, 0–5%, 5–10%, 10–20%, 20–30%, 30–40%, 40–60% and 60–80%, for ${\bar{\Xi }}^{+}$, 0–5%, 5–10%, 10–20%, 20–40%, 40–60% and 60–80%, for ${\bar{\Omega }}^{+}$, 0–20%, 20–40% and 40–60% at |y|<0.1, and for deuteron (*d*), 0–10%, 10–20%, 20–40%, 40–60% and 60–80%, at |y|<0.3. The symbols are cited from the experimental data measured by the STAR Collaboration at the Relativistic Heavy Ion Collider (RHIC) [21,55,56]. In the figure, the curves are our fitted results from Equation (1). The corresponding values of the free parameters ($T_{0}$, *V*, $\beta_{T}$ and $n_{0}$), normalization constant $\left(N_{0}\right)$, $\chi^{2}$ and number of degrees of freedom (ndof) are listed in Table 1, the parameter trend of which is analyzed and discussed later in this section. One can see that the $p_{T}$ spectra of the particles are shown to obey approximately the blast wave model with Boltzmann Gibbs statistics. Furthermore, the spectra of $\pi^{+}$ in 5–10%, 10–20%, 20–30%, 30–40%, 40–50%, 50–60%, 60–70% and 70–80% centrality intervals are scaled with 1/3, 1/7, 1/18, 1/40, 1/100, 1/250, 1/700 and 1/1500 respectively, while the centrality intervals 5–10%, 10–20%, 20–30%, 30–40%, 40–50%, 50–60%, 60–70% and 70–80% of *p* are scaled by 1/3, 1/7, 1/26, 1/60, 1/150, 1/250, 1/400 and 1/600, respectively.

**Figure 2:**
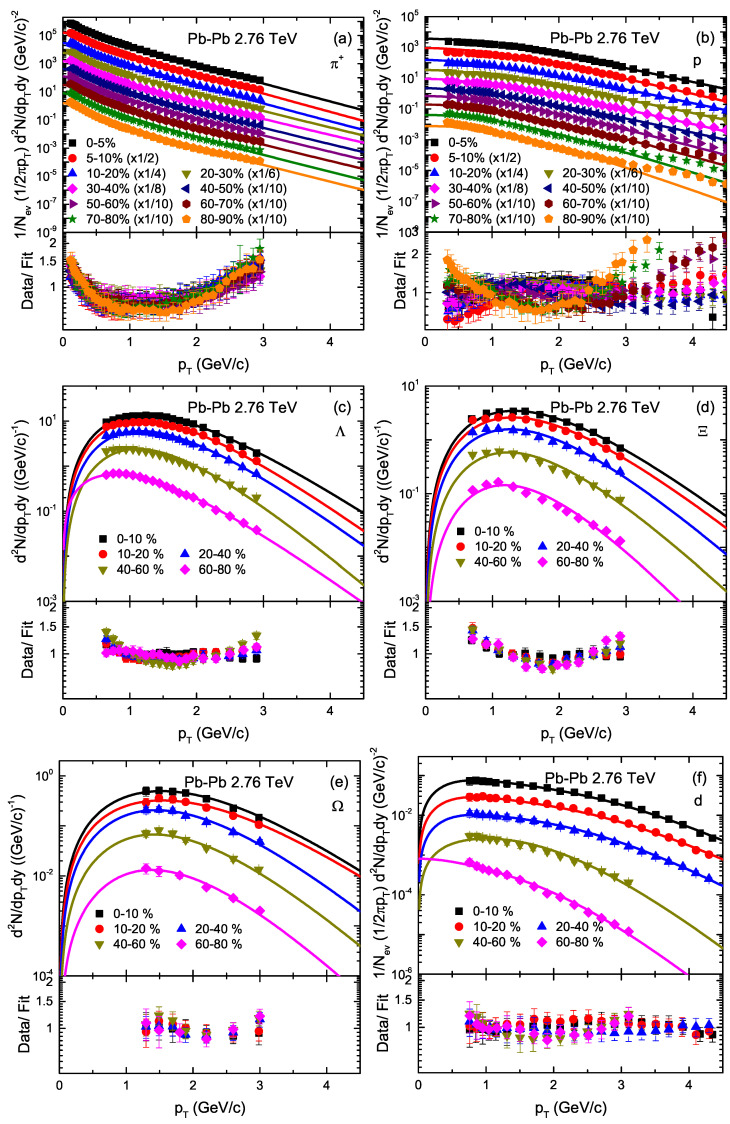
Transverse momentum spectra of $\pi^{+}$, *p*, Λ, Ξ, Ω and deuteron (d) produced in different centrality intervals in Pb–Pb collisions at 2.76 TeV at rapidity |y|<0.5. Different symbols represent the $p_{T}$ spectra of different particles measured by the ALICE collaboration [57,58,59] and the curves are our fitted results with the BGBW model. The corresponding results of the data/fit are presented in each panel.

Figure 2 is similar to Figure 1, but it shows the the $p_{T}$ spectra of $\pi^{+}$, *p*, Λ, Ξ, Ω and deuteron (d) in different centrality intervals in Pb–Pb collisions at 2.76 TeV. The spectra are distributed in different centrality intervals; e.g., for $\pi^{+}$, and *p*; 0–5%, 5–10%, 10–20%, 20–30%, 30–40%, 40–50%, 50–60%, 60–70% 70–80% and 80–90% at |y|<0.5, for Λ, Ξ, and Ω; 0–10%, 10–20%, 20–40%, 40–60% and 60–80%, for Ω; 0–10%, 10–20%, 20–40%, 40–60% and 60–80% at y=0, and for deuteron (*d*); 0–10%, 10–20%, 20–40%, 40–60% and 60–80%, at |y|<0.5. The spectra of $\pi^{+}$ and *p* in 5–10%, 10–20%, 20–30%, 30–40%, 40–50%, 50–60%, 60–70% and 70–80% centrality intervals are scaled with 1/2, 1/4, 1/6, 1/8, 1/10, 1/10, 1/10, 1/10 and 1/10, respectively. The symbols are cited from the experimental data measured by the ALICE Collaboration at the Large Hadron Collider (LHC) [57,58,59]. In the figure, the curves are our fitted results with a result of 231 (1). The corresponding values of free parameters ($T_{0}$, *V*, $\beta_{T}$ and $n_{0}$), normalization constant $\left(N_{0}\right)$, $\chi^{2}$ and number of degrees of freedom (ndof) are listed in Table 1, the parameter trend of which is analyzed and discussed below. One can see that the $p_{T}$ spectra of the particles are shown to obey approximately the blast wave model with Boltzmann Gibbs statistics. Note that we have used the method of least squares to obtain the parameters in the present work, and the fits (especially the ALICE data) to the BGBW model are not good for quite abundant hadron species, such as $\pi^{+}$ and protons, due to the generation of non-inclusion resonance in the low $p_{T}$ region. In addition, we would also like to point out that the values of $\chi^{2}$ vary, especially in some cases in central collisions, where it increases due to poor fitting.

**Figure 3:**
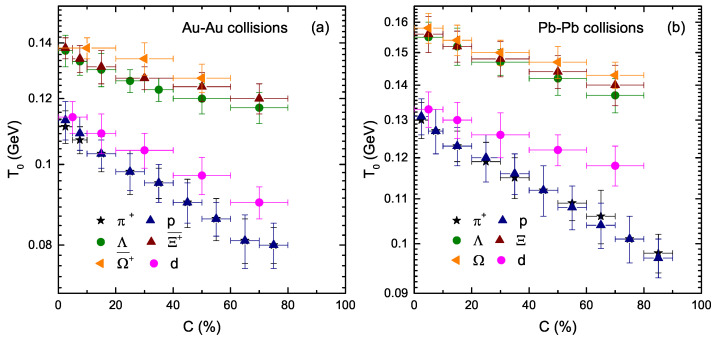
Dependence of $T_{0}$ on the centrality class (C%) and resting mass ($m_{0}$) of the particle.

Figure 3 shows the dependence of the kinetic freezeout temperature ($T_{0}$) on the centrality class (C%) and mass of the particles. Panels (a) and (b) show the results for Au–Au and Pb–Pb collisions, respectively. The colored symbols represent different species of particles, and the particles from left to right show the result of $T_{0}$ from central to peripheral collisions. One can see that the kinetic freezeout temperature of the emission source decreases with the decrease of centrality from central to peripheral collisions. The central collision corresponds to a very violent collision due to the large number of participant nucleons, which makes the degree of excitation of the system high and results in a high temperature, but as the centrality decreases, the collision become decreasingly violent due to the small number of particles involved in the interaction, which results in the degree of excitation of the system decreasing, and correspondingly the temperature decreases. This result is consistent with [5,6,18,27,28,29,60], but inconsistent with [61,62,63,64,65]. In addition, the dependence of $T_{0}$ on $m_{0}$ is not clear. The pion and proton have almost the same values for $T_{0}$, and similarly the strange (muti-strange) particles have almost the same values for $T_{0}$. Deuteron has the largest mass, and it freezes out at the same time as the pion and proton. The reason may be the production cross-section of the interacting particle. According to kinematics, the reactions with a smaller cross-section are supposed to be switched-off at higher temperatures/densities or earlier in time than the reactions with larger cross-sections. $\pi^{+}$, *p* and *d* are non-strange particles, so they have the same $T_{0}$, while Λ, Ξ(${\bar{\Xi }}^{+}$) and Ω(${\bar{\Omega }}^{+}$) are strange-flavored particles, so they have the same $T_{0}$. The non-strange particles have a larger production cross-section than the strange or multi-strange particles; therefore, the non-strange particles freezeout later than the strange (multi-strange) particles. This result is consistent with that of our recent work [10]; however, in [10], the authors also observed a separate decoupling of strange and multi-strange particles. It is noteworthy that the observed $T_{0}$ at the RHIC is lower than that of the LHC. In addition, we would also like to point out that several previous works have studied the fit of the blast wave with different methods and obtained different results from those of our recent work. In the present work, the least square method is used, and we observed the double kinetic freezeout scenario, while the previous literature observed single or multiple kinetic freezeout scenarios.

**Figure 4:**
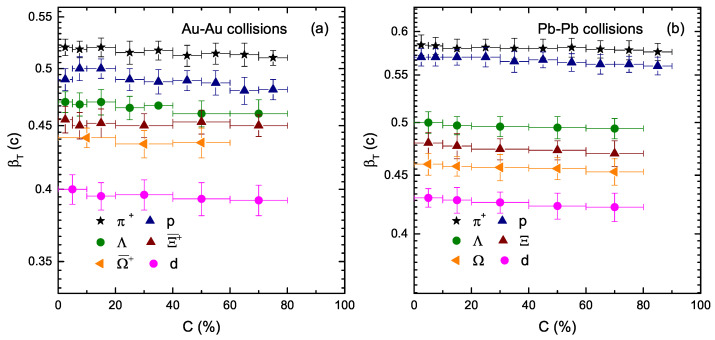
Dependence of $\beta_{T}$ on the centrality class (C%) and resting mass ($m_{0}$) of the particle.

Figure 4 is similar to Figure 3, but shows the dependence of the transverse flow velocity ($\beta_{T}$) on the centrality class and mass of the particles. One can see that $\beta_{T}$ depends on the resting mass of the particles. The greater the mass of the particle, the smaller the transverse flow velocity. In fact, some hydrodynamic simulations observed the same velocity for the flow of all the particles, but they presented different explanations. Besides, different models give different results. The selection of $\beta_{T}$ is more technical and complex; in some cases, it even depends on the range of $p_{T}$, such that the selections of ranges are different for different models. Furthermore, there is no centrality dependence of $\beta_{T}$ observed in the present work, as $\beta_{T}$ is almost the same in the central and peripheral collisions. The reason behind this is that the collective behavior at the stage of kinetic freezeout does not change from central to peripheral collisions. However, $\beta_{T}$ is larger at the LHC than that of the RHIC.

**Figure 5:**
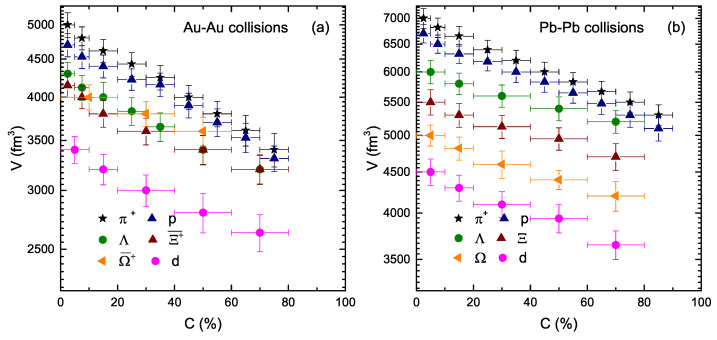
Dependence of *V* on the centrality class (C%) and resting mass ($m_{0}$) of the particle.

Figure 5 is similar to Figure 3 and Figure 4 but shows the dependence of *V* on the centrality class and mass of the particles. One can see that *V* decreases continuously from central to peripheral collisions because the central collisions correspond to a large number of binary collisions due to the re-scattering of partons, and hence the system with more participants quickly reaches the equilibrium state, while the number of participants decreases with the decrease of event centrality and the system reaches an equilibrium state in a steady manner from central to peripheral collisions. Additionally, *V* depends on the mass of the particles. The greater the mass of the particle, the lower the *V*. *V* at the LHC is larger than that at the RHIC.

It should be noted that the cases of $T_{0}$ and/or $\beta_{T}$ are very complex on the basis of their dependence on centrality. The observed results can be changed by changing the model, by using the same model but a different method or by changing the limits and conditions of the model, such that by changing the parameters, we can get different results. For example, if for central collisions, one use a smaller $T_{0}$ and a larger $\beta_{T}$, a decreasing trend for $T_{0}$ from peripheral to central collisions can be obtained. At the same time, a negative correlation between $T_{0}$ and $\beta_{T}$ will also be obtained. Similarly, if one use a larger $T_{0}$ and a smaller $\beta_{T}$, an increasing trend for $T_{0}$ from peripheral to central collisions can be obtained. At the same time, a positive correlation between $T_{0}$ and $\beta_{T}$ will also be obtained.

## 4. Conclusions

The main observations and conclusions of our work are summarized here.

(a) The transverse momentum spectra of different particle species are analyzed by the blast wave model with Boltzmann Gibbs statistics, and the bulk properties in terms of the kinetic freezeout temperature, transverse flow velocity and freezeout volume are extracted in different centrality classes in nucleus–nuclues collisions at center of mass energy.
(b) It is observed that $T_{0}$ is dependent on the cross-section of the interacting particle; i.e., a larger production cross-section of the interacting particle corresponds to a smaller $T_{0}$.
(c) A double kinetic freezeout scenario is observed due to the separate decoupling of non-strange and strange (multi-strange) particles.
(d) The transverse flow velocity ($\beta_{T}$) and kinetic freezeout volume (*V*) are observed to depend on the mass of the particles; i.e., the larger the mass of the particle, the smaller the $\beta_{T}$ and *V*.
(e) The kinetic freezeout temperature ($T_{0}$) and freezeout volume (*V*) decrease from central peripheral collisions due to the decrease of the degree of excitation of the interacting system and the decrease of the number of binary collisions due to the re-scattering of partons from central to peripheral collisions, respectively. At the same time, $\beta_{T}$ is observed to be independent of centrality and remains almost unchanged from central to peripheral collisions because the collective behavior at the stage of the kinetic freezeout in the interacting system does not change with event centrality.
(f) $T_{0}$, $\beta_{T}$ and *V* are observed to be larger for collisions at the LHC that at the RHIC.
(g) The obtained results can be changed by changing the model, by using the same model with a different method or by changing the parameters used in the model.

## Figures and Tables

**Table 1:** Values of free parameters ($T_{0}$ and $\beta_{T}$, V, normalization constant ($N_{0}$), $n_{0}$), $\chi^{2}$ and degree of freedom (dof) corresponding to the curves in Figure 1 and Figure 2.

Collisions	Centrality	Particle	$T_{0}$ (GeV)	$\beta_{T}$ (c)	$V({\mathrm{fm}}^{3})$	$N_{0}$	$n_{0}$	$\chi^{2}$/dof
Figure 1	0–5%	$\pi^{+}$	0.111±0.005	0.520±0.008	5000±193	0.25±0.06	0.8	3/5
Au–Au	5–10%	–	0.107±0.004	0.518±0.008	4800±170	0.24±0.004	1.3	7/5
62.4 GeV	10–20%	–	0.103±0.005	0.520±0.009	4615±165	0.185±0.004	2.6	2/5
	20–30%	–	0.098±0.006	0.515±0.011	4430±161	0.136±0.0005	1.3	2/5
	30–40%	–	0.095±0.004	0.517±0.009	4250±160	0.0975±0.004	1.2	2/5
	40–50%	–	0.090±0.006	0.512±0.010	4000±150	0.067±0.004	1.8	5/5
	50–60%	–	0.086±0.005	0.514±0.010	3800±150	0.049±0.005	2	7/5
	60–70%	–	0.081±0.005	0.513±0.011	3610±170	0.029±0.004	2	1/5
	70–80%	–	0.080±0.004	0.510±0.007	3400±176	0.015±0.005	2	4/5
Figure 1	0–5%	*p*	0.113±0.006	0.490±0.010	4700±170	0.0165±0.003	1.2	33/9
Au–Au	5–10%	–	0.109±0.005	0.500±0.011	4530±160	0.0094±0.0005	1	20/9
62.4 GeV	10–20%	–	0.105±0.004	0.500±0.009	4400±155	0.0113±0.004	1.2	5/9
	20–30%	–	0.100±0.005	0.490±0.010	4225±140	0.008±0.0005	1.3	3/9
	30–40%	–	0.097±0.005	0.488±0.011	4160±150	0.0055±0.0004	1.5	7/9
	40–50%	–	0.093±0.005	0.489±0.009	3900±150	0.0035±0.0004	0.8	3/9
	50–60%	–	0.088±0.004	0.487±0.011	3700±158	0.0022±0.0003	0.6	4/9
	60–70%	–	0.083±0.006	0.480±0.012	3530±160	0.00175±0.0004	0.3	4/9
	70–80%	–	0.081±0.005	0.481±0.009	3310±130	0.00055±0.00005	0.4	14/9
Figure 1	0–5%	Λ	0.137±0.006	0.470±0.009	4300±152	0.023±0.004	0.7	1/7
Au–Au	5–10%	–	0.133±0.005	0.468±0.010	4120±160	0.002±0.0004	0.7	1/7
62.4 GeV	10–20%	–	0.130±0.006	0.470±0.011	4000±187	0.00017±0.00004	0.7	1/7
	20–30%	–	0.126±0.004	0.465±0.010	3830±164	$1\times 10^{-5}\pm 4\times 10^{-6}$	0.8	1/7
	30–40%	–	0.123±0.004	0.467±0.012	3650±160	$9\times 10^{-7}\pm 5\times 10^{-8}$	0.8	1/7
	40–60%	–	0.120±0.005	0.460±0.011	3400±156	$4\times 10^{-8}\pm 3\times 10^{-9}$	0.8	5/7
	60–80%	–	0.117±0.005	0.460±0.012	3200±140	$1\times 10^{-9}\pm 5\times 10^{-10}$	0.9	5/7
Figure 1	0–5%	${\bar{\Xi }}^{+}$	0.138±0.004	0.455±0.011	4150±150	0.0008±0.00004	0.7	0.4/5
Au–Au	5–10%	–	0.134±0.005	0.450±0.011	4000±140	$6.5\times 10^{-5}\pm 6\times 10^{-6}$	1	3/6
62.4 GeV	10–20%	–	0.131±0.006	0.452±0.012	3800±157	$5.2\times 10^{-6}\pm 4\times 10^{-7}$	0.8	2/6
	20–40%	–	0.127±0.004	0.450±0.010	3600±148	$4.5\times 10^{-7}\pm 6\times 10^{-8}$	0.7	3/6
	40–60%	–	0.124±0.005	0.453±0.010	3400±150	$8.8\times 10^{-9}\pm 5\times 10^{-10}$	0.7	1/6
	60–80%	–	0.120±0.005	0.450±0.009	3200±146	$3.4\times 10^{-10}\pm 5\times 10^{-11}$	0.4	3/4
Figure 1	0–20%	${\bar{\Omega }}^{+}$	0.138±0.004	0.440±0.008	4000±155	$5.2\times 10^{-5}\pm 5\times 10^{-6}$	0.6	0.3/0
Au–Au	20–40%	–	0.134±0.006	0.435±0.011	3800±146	$2\times 10^{-7}\pm 6\times 10^{-8}$	1	1/0
62.4 GeV	40–60%	–	0.127±0.005	0.436±0.012	3600±160	$3.2\times 10^{-9}\pm 7\times 10^{-10}$	0.7	2/−1
Figure 1	0–10%	*d*	0.114±0.005	0.400±0.011	3400±140	0.00085±0.00005	1.6	3/7
Au–Au	10–20%	–	0.109±0.006	0.395±0.010	3200±150	0.00034±0.00004	1.6	2/7
62.4 GeV	20–40%	–	0.104±0.005	0.396±0.011	3000±145	0.0001±0.00004	1.5	1/7
	40–60%	–	0.097±0.005	0.393±0.012	2800±170	$2\times 10^{-5}\pm 5\times 10^{-6}$	1.3	1/7
	60–80%	–	0.090±0.004	0.392±0.011	2632±150	$2\times 10^{-6}\pm 4\times 10^{-7}$	0.9	22/6
Figure 2	0–5%	$\pi^{+}$	0.130±0.005	0.584±0.012	7000±200	345±36	0.8	89/36
Pb–Pb	5–10%	–	0.127±0.006	0.583±0.010	6816±191	165.55±23	0.7	158/36
2.76 TeV	10–20%	–	0.123±0.004	0.580±0.011	6650±185	60.55±8	0.8	93/36
	20–30%	–	0.119±0.005	0.581±0.010	6392±180	18.80±3	0.9	58/36
	30–40%	–	0.115±0.005	0.580±0.012	6200±185	6.3±0.4	1	54/36
	40–50%	–	0.112±0.006	0.580±0.011	6000±170	2.2±0.3	1	92/36
	50–60%	–	0.109±0.004	0.581±0.011	5843±162	0.66±0.04	1	100/36
	60–70%	–	0.106±0.006	0.579±0.010	5670±170	0.16±0.03	1.1	197/36
	70–80%	–	0.101±0.005	0.578±0.011	5500±166	0.04±0.005	1.1	151/36
	80–90%	–	0.098±0.004	0.576±0.010	5300±160	0.008±0.0004	1.1	221/36
Figure 2	0–5%	*p*	0.131±0.005	0.570±0.010	6700±180	8±0.7	1	58/30
Pb–Pb	5–10%	–	0.127±0.006	0.570±0.010	6500±170	4.05±0.5	0.9	125/37
2.76 TeV	10–20%	–	0.123±0.005	0.570±0.009	6320±170	1.35±0.3	1.1	37/33
	20–30%	–	0.120±0.006	0.570±0.011	6180±160	0.9±0.05	1.1	34/31
	30–40%	–	0.116±0.005	0.565±0.012	6000±180	0.16±0.04	1.07	17/31
	40–50%	–	0.112±0.006	0.567±0.009	5830±170	0.05±0.004	1.1	108/33
	50–60%	–	0.108±0.005	0.564±0.010	5650±165	0.016±0.003	1	62/31
	60–70%	–	0.104±0.005	0.562±0.011	5480±170	0.0045±0.0004	1	140/34
	70–80%	–	0.101±0.005	0.562±0.009	5300±180	0.001±0.0003	0.9	214/36
	80–90%	–	0.097±0.004	0.560±0.010	5100±180	0.0002±0.00003	0.8	207/37
Figure 2	0–10%	Λ	0.155±0.005	0.500±0.011	6000±200	0.13±0.03	0.9	28/14
Pb–Pb	10–20%	–	0.152±0.006	0.497±0.009	5800±180	0.1±0.03	0.8	27/14
2.76 TeV	20–40%	–	0.147±0.004	0.496±0.010	5600±180	0.06±0.004	0.8	35/14
	40–60%	–	0.142±0.005	0.495±0.011	5400±185	0.024±0.004	0.6	124/14
	60–80%	–	0.137±0.005	0.494±0.010	5200±170	0.0074±0.0005	1.1	17/14
Figure 2	0–10%	Ξ	0.156±0.006	0.480±0.010	5500±200	0.0180±0.004	1	16/7
Pb–Pb	10–20%	–	0.152±0.005	0.477±0.012	5300±180	0.0140±0.003	1	37/7
2.76 TeV	20–40%	–	0.148±0.005	0.474±0.010	5126±170	0.0085±40.0005	0.9	63/7
	40–60%	–	0.144±0.005	0.473±0.009	4950±160	0.0032±0.0005	0.8	82/7
	60–80%	–	0.140±0.006	0.470±0.012	4700±180	0.0008±0.00005	0.6	107/7
Figure 2	0–10%	Ω	0.158±0.005	0.460±0.010	5000±150	0.0014±0.0003	1.1	12/2
Pb–Pb	10–20%	–	0.154±0.005	0.458±0.009	4817±160	$9.7\times 10^{-4}\pm 4\times 10^{-5}$	1.2	1/2
2.76 TeV	20–40%	–	0.150±0.004	0.457±0.012	4600±180	$6\times 10^{-4}\pm 6\times 10^{-5}$	0.9	3/2
	40–60%	–	0.147±0.005	0.456±0.010	4400±120	$2\times 10^{-4}\pm 5\times 10^{-5}$	0.8	6/2
	60–80%	–	0.143±0.004	0.453±0.012	4200±180	$4\times 10^{-5}\pm 5\times 10^{-6}$	0.7	5/1
Figure 2	0–10%	*d*	0.133±0.005	0.430±0.008	4500±170	$4.6\times 10^{-4}\pm 5\times 10^{-5}$	1.8	6/16
Pb–Pb	10–20%	–	0.130±0.005	0.428±0.011	4300±160	$1.8\times 10^{-4}\pm 4\times 10^{-5}$	1.8	6/16
2.76 TeV	20–40%	–	0.126±0.006	0.426±0.009	4100±153	$6.6\times 10^{-5}\pm 4\times 10^{-6}$	1.7	4/16
	40–60%	–	0.122±0.004	0.423±0.011	3938±160	$1.5\times 10^{-5}\pm 5\times 10^{-6}$	1.2	15/10
	60–80%	–	0.118±0.005	0.422±0.012	3650±150	$2.8\times 10^{-6}\pm 4\times 10^{-7}$	1.3	10/10

## Data Availability

The data used to support the findings of this study are included within the article and are cited at relevant places within the text as references.
